# Plants as highly diverse sources of construction wood, handicrafts and fibre in the Heihe valley (Qinling Mountains, Shaanxi, China): the importance of minor forest products

**DOI:** 10.1186/s13002-017-0165-8

**Published:** 2017-06-30

**Authors:** Jin Kang, Yongxiang Kang, Jing Feng, Mengying Liu, Xiaolian Ji, Dengwu Li, Kinga Stawarczyk, Łukasz Łuczaj

**Affiliations:** 1 College of Forestry, Northwest A&F University, 712100 Yangling, Shaanxi People’s Republic of China; 2 Yangling Vocational & Technical College, 712100 Yangling, Shaanxi People’s Republic of China; 3 College of Landscape Architecture and Arts, Northwest A&F University, 712100 Yangling, Shaanxi People’s Republic of China; 4 Department of Botany, Institute of Biotechnology, University of Rzeszów, Werynia 502, 36-100 Kolbuszowa, Poland

**Keywords:** Minor timber forest products, Non-timber forest products, Taibai

## Abstract

**Background:**

Chinese rural communities living among species-rich forests have little documentation on species used to make handicrafts and construction materials originating from the surrounding vegetation. Our research aimed at recording minor wood uses in the Heihe valley in the Qinling mountains.

**Methods:**

We carried out 37 semi-structured interviews in seven villages.

**Results:**

We documented the use of 84 species of plants. All local large canopy trees are used for some purpose. Smaller trees and shrubs which are particularly hard are selectively cut. The bark of a few species was used to make shoes, hats, steamers and ropes, but this tradition is nearly gone. A few species, mainly bamboo, are used for basket making, and year-old willow branches are used for brushing off the chaff during wheat winnowing.

**Conclusions:**

The traditional use of wood materials documented suggests that some rare and endangered tree species may have been selectively cut due to their valuable wood, e.g. *Fraxinus mandshurica* and *Taxus wallichiana* var. *chinensis*. Some other rare species, e.g. *Dipteronia sinensis,* are little used and little valued.

## Background

Construction wood and firewood are the main products of modern forestry. However local communities living in woodlands usually implement multiple uses of the forest, also involving the production of utensils, medicine and food. The importance of minor timber forest products and non-timber forest products (NTFP) has been emphasized for decades in ethnobotany, forestry, rural development etc. Some of these products may have a vital non-commercial value,others enter the cash economy and improve livelihoods [1–6]. Ethnobotanical works, however, often overlook the lesser-used types of wood available to local populations, emphasizing only the “non-timber” part of the ecosystem. The minor uses of wood are more closely documented in older ethnographic works. e.g. describing and documenting traditional tools and handicrafts, although the topic has also been touched upon by ethnobotany [7–14].

Chinese ethnobotany has been developing fast in recent years. However most papers are focused on traditional wild food and medicine, mainly among ethnic minorities. Although some papers are devoted to the issue of non-timber forest products in China [15–19], we observed a lack of studies concerning the ethnobotany of traditional handicrafts and other objects made of wood. In order to fill this gap we carried out a study in the Heihe National Forest Park in the Taibai range, Shaanxi province, China. Mount Taibai, the highest of the Qinling Mountains, is one of the most species-rich and valuable parts of nature in northern China. This area has preserved a rich woodland flora and fauna, which is well-studied. An area with a rich and well-documented flora is an ideal working place for an ethnobotanist. Over the past few years some of the authors of this paper have devoted a few articles to the use of wild food plants in one of the valleys of the Taibai range, and the use and cultivation of the highly toxic *Aconitum carmichaelii* [20–22].

Our research aimed to document minor wood uses in the Heihe valley. By this we mean any uses of wood, twigs or branches of trees, shrubs, climbers and bamboo apart from large scale construction wood or firewood. Both past uses (before the area became a national forest park) and present uses were recorded.

## Methods

### Study area

**Fig. 1:**
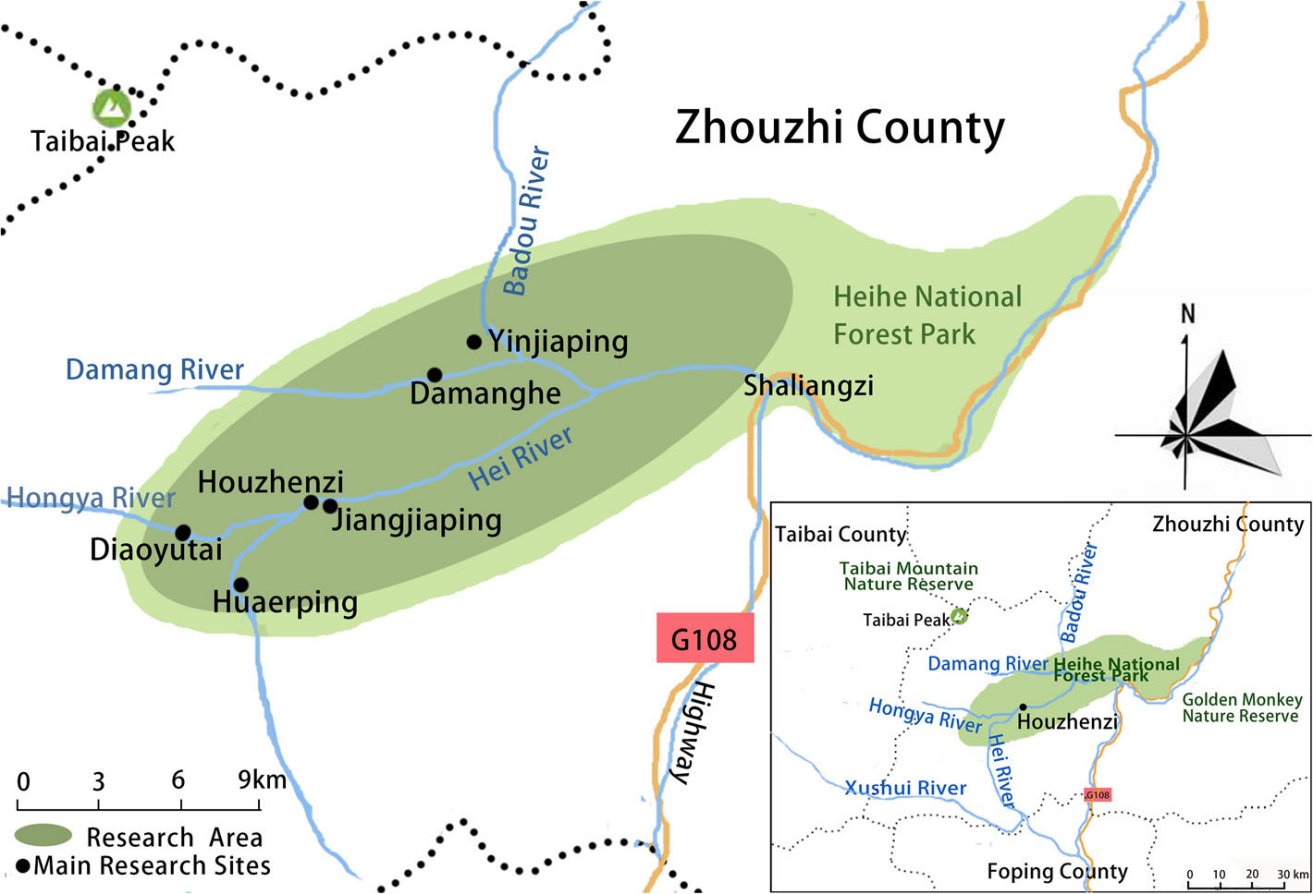
The location of the study

The study covers the Heihe National Forest Park (Fig. 1), on the southern side of the Taibai Nature Reserve, with the highest peak of northern China in the center of the reserve (Mt Taibai 3767 m a.s.l.). The nature reserve protects a highly diverse flora – from warm temperate (with subtropical elements), to alpine at the top. The National Forest Park (with a less strict protection regime) is adjacent to it, and mainly protects species-rich forests. The area is almost completely covered by ancient forest vegetation and rocky outcrops. The Heihe river valley belongs to the Houzhenzi administrative unit (town, *zhen* (镇)), with an area of 822 km^2^. It is a very isolated place, which has vehicular access to the county town of Zhouzhi (where the post-office and schools are located) only via a 2.5 h drive through a winding precipitous gorge, sometimes blocked for days by falling rocks. The whole valley is inhabited by 2813 people [23] – a quarter of them in the main settlement of Houzhenzi, and the rest in hamlets scattered throughout the forest (Fig. 1).

The studied villages lie between 1000 and 1500 m a.s.l. At these altitudes the climate is temperate, with daily temperatures in summer oscillating around 20–30 °C and winter temperatures around 10 °C to – 10 °C. The mean annual temperature in Houzhenzi is 8.2 °C, with a high rainfall of nearly 1000 mm, 44% of which is concentrated in the summer months. The dominant vegetation is the species-rich *Quercus variabilis* and *Q. aliena* var. *acuteserrata* forest, with an admixture of *Pinus tabuliformis,* and many deciduous tree species (e.g. *Acer* spp., *Tilia* spp.).

The majority of the local population are subsistence Han Chinese farmers who grow maize, potatoes, wheat and beans. Sources of cash income are the orchards of zaopi (*Cornus officinalis*), walnuts (*Juglans regia*) and northern Sichuan pepper (*Zanthoxylum bungeanum*). Digging out medicinal roots and collecting medicinal herbs for wholesale buyers is also a very popular activity. The importance of tourism is increasing. A significant proportion of farms are registered as agritourist farms (*nong jia le*). Most tourists come from Xian and its surroundings and are attracted by the beautiful scenery and hiking opportunities.

### Data collection

The field research was conducted in the summer and autumn of 2016 using the Rapid Rural Appraisal approach [24, 25], and included 37 freelisting interviews in seven villages (Fig. 1), which involved 52 people altogether. This included 39 men and 13 women as the former were more willing to talk about this topic. The mean age of the participants was 55 (aged from 39 to 87).

**Fig. 2:**
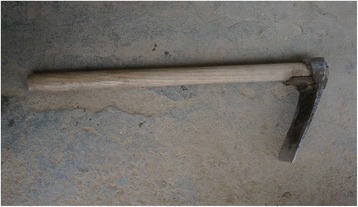
A narrow hoe *(juetou* 镢头) resembling a pick-axe is a common agricultural tool, very useful in stoney mountain soil. The handle was made from a *Cornus kousa* branch

**Fig. 3:**
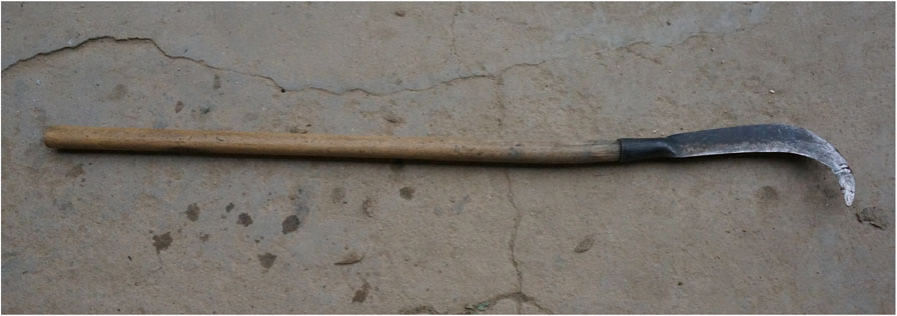
A sickle on a long handle (liandao, 镰刀, this one made of *Cornus kousa*) is another indispensable tool in the area

**Fig. 4:**
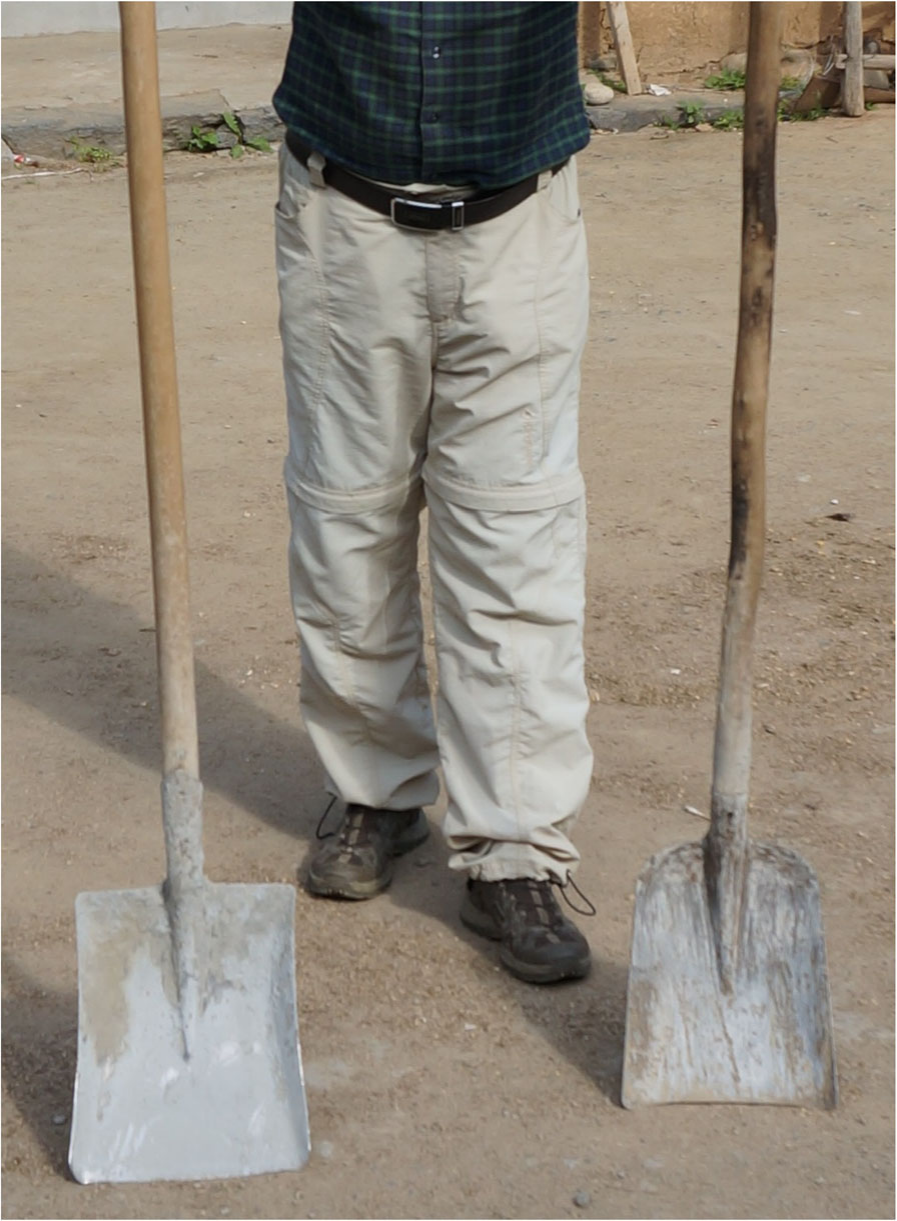
Two spade handles – the one on the left made from *Meliosma* wood, the one on the right from *C. kousa*

**Fig. 5:**
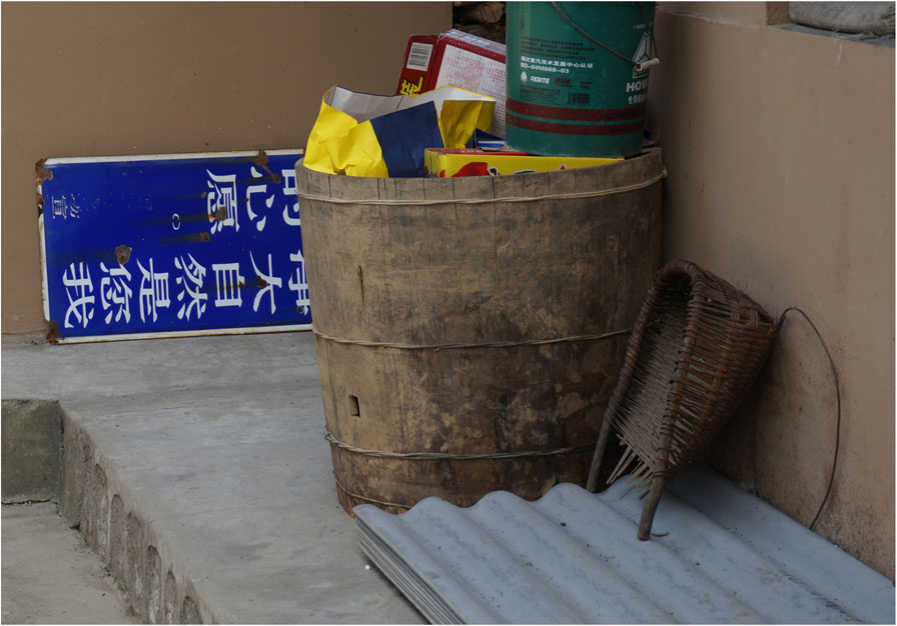
A barrel made of *Catalpa* wood

**Fig. 6:**
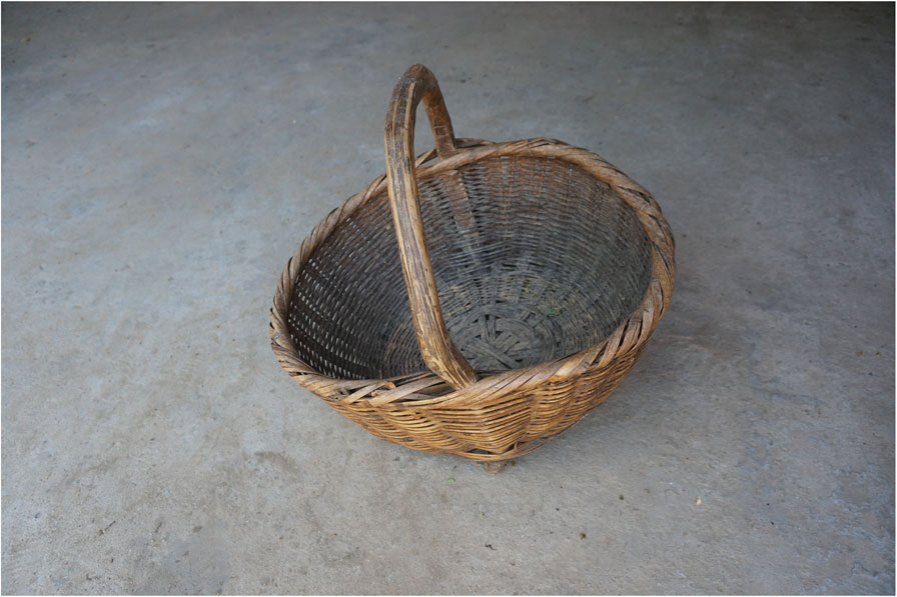
The commonest type of basket made of *Phyllostachys* bamboo. The handle was made of *C. kousa*

**Fig. 7:**
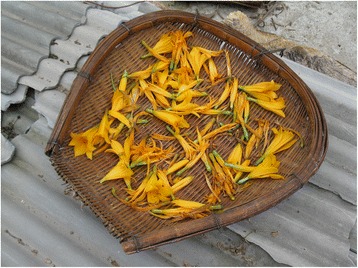
Bamboo trays are commonly used to dry plants for winter

**Fig. 8:**
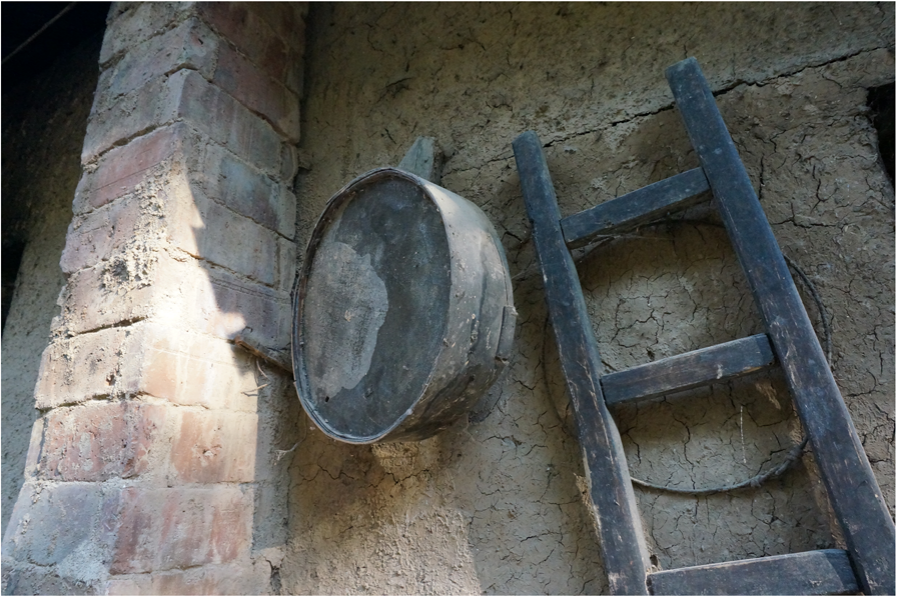
Sieve walls are made of *Betula albosinensis* wood

**Fig. 9:**
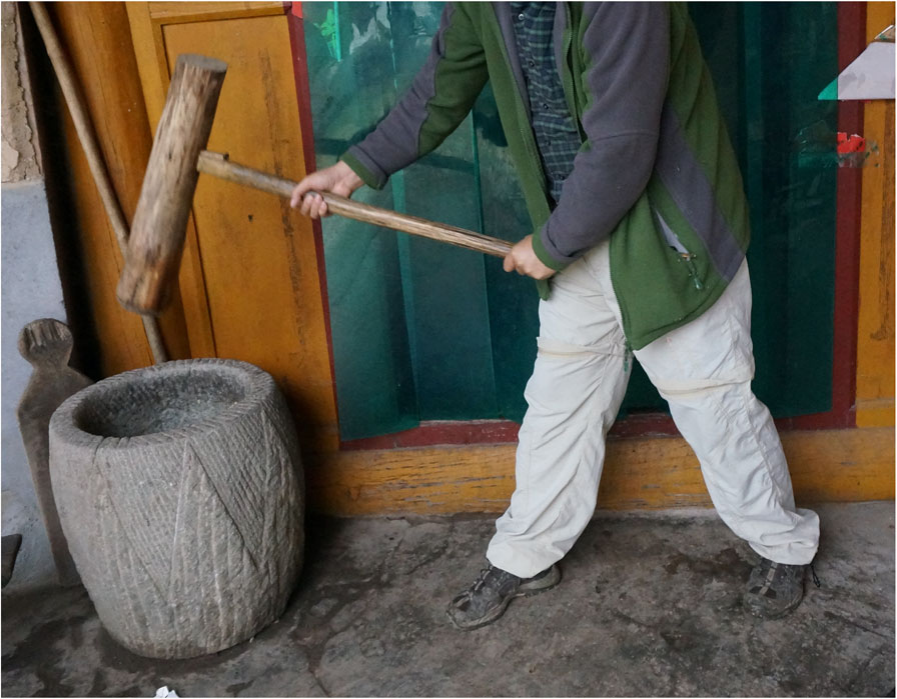
A *ciba* hammer used for pounding some foodstuffs

**Fig. 10:**
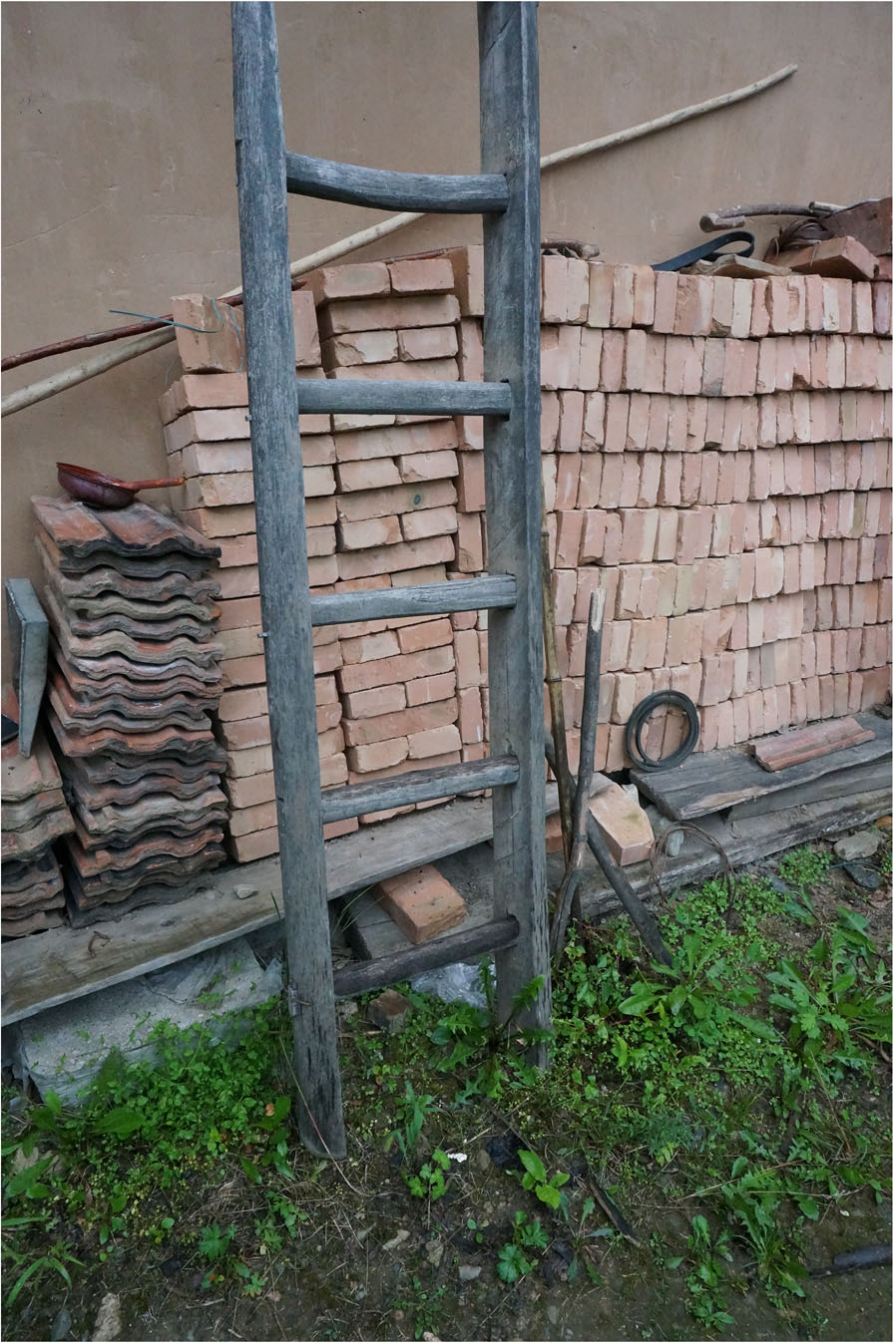
A ladder made of *Tilia*

**Fig. 11:**
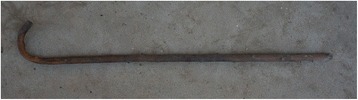
A walking stick made of *Berchemia sinica*

**Fig. 12:**
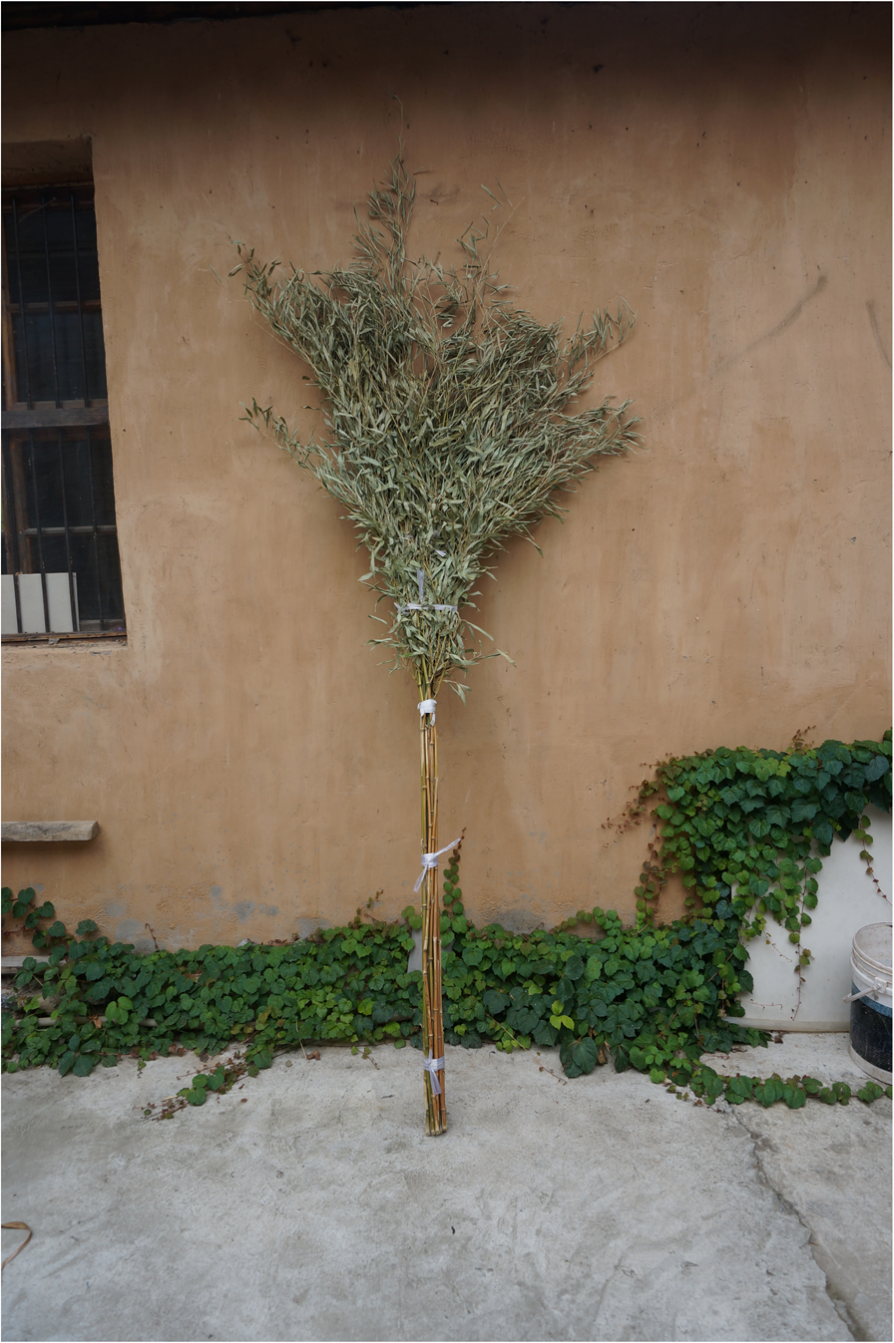
A broom made of locally grown *Phyllostachys* bamboo

**Fig. 13:**
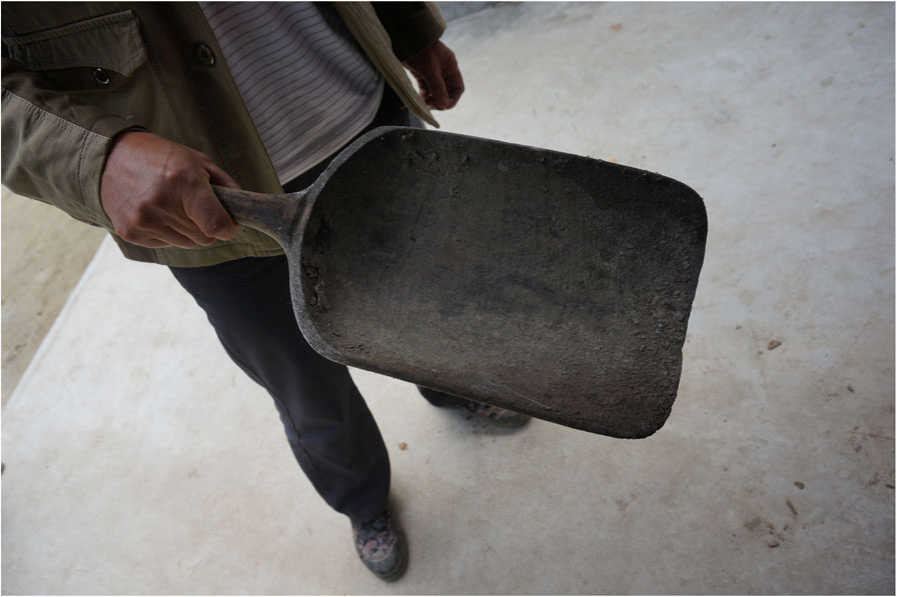
A *cuopiao* grain shovel made of *Populus purdomii*

**Fig. 14:**
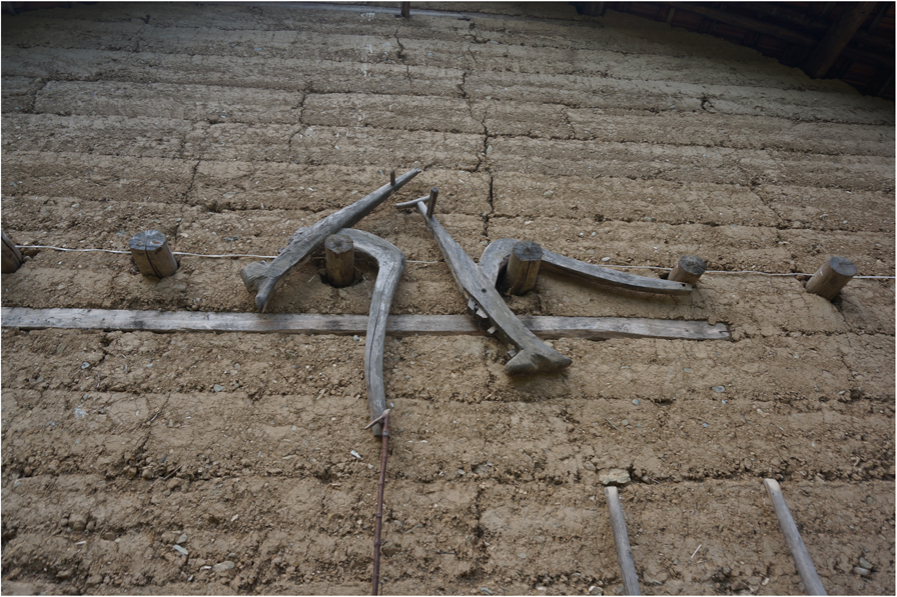
A plough made of *Ulmus* wood

**Fig. 15:**
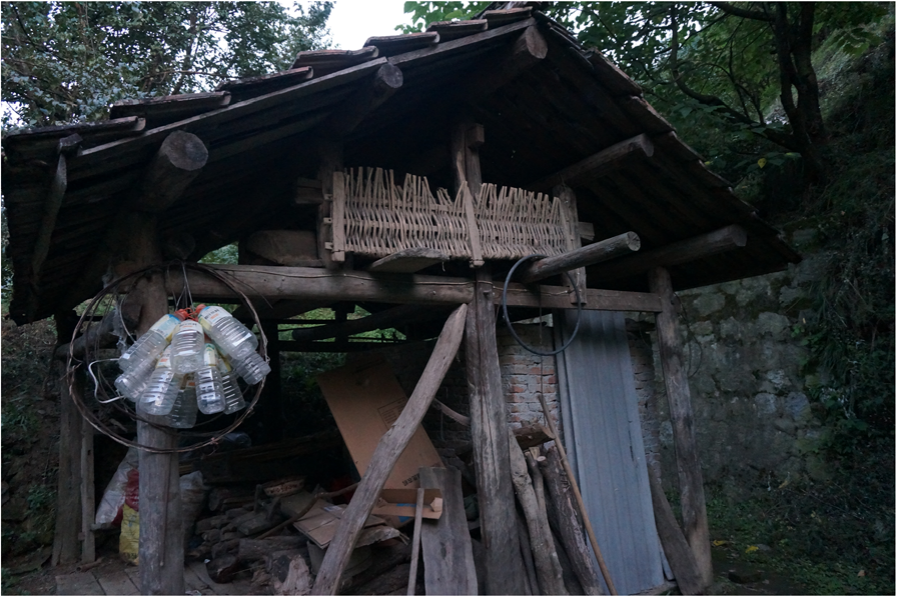
A harrow (*mu*) with ‘teeth” made of *Cotinus* wood

**Fig. 16:**
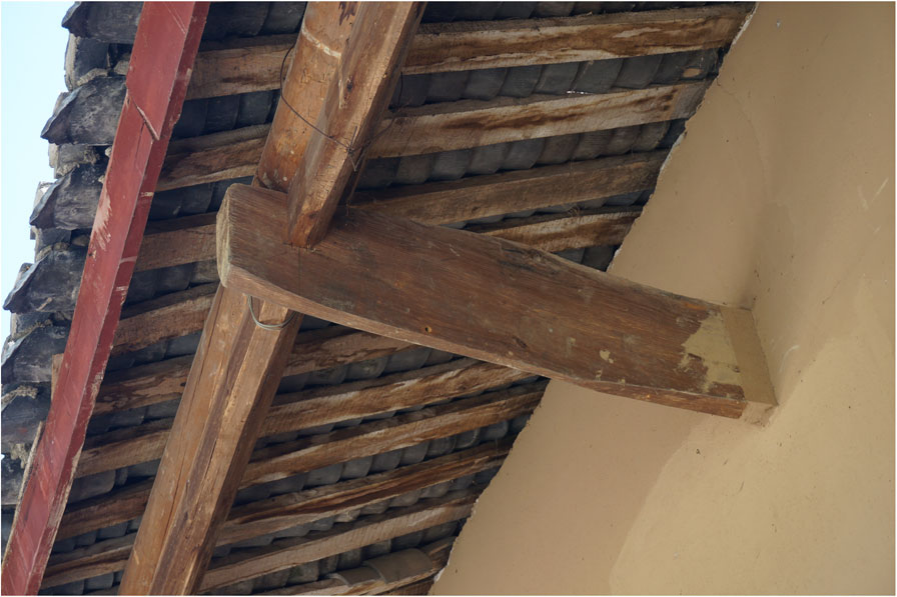
Boards supporting tiles are often made of *Toxicondendron vernicifluum* wood

**Fig. 17:**
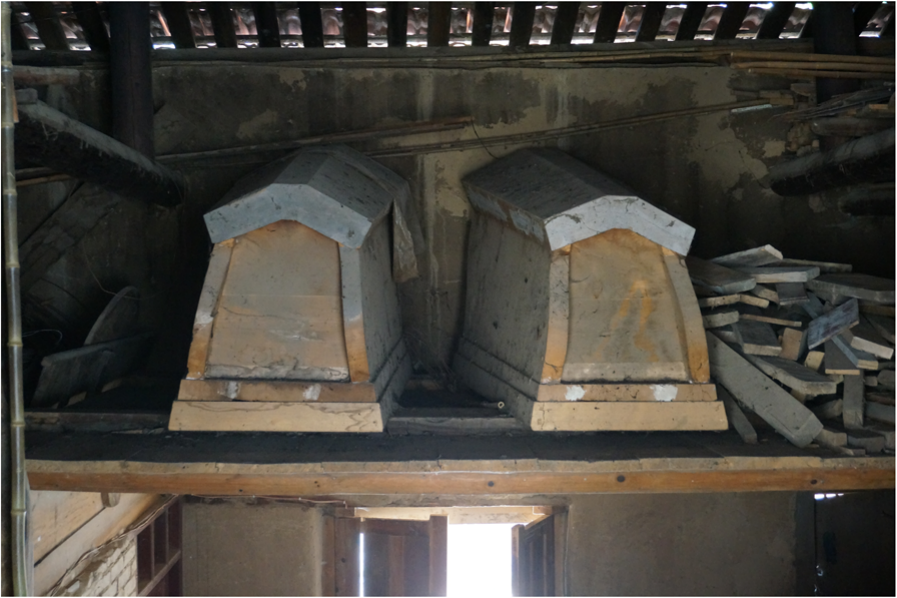
Coffins are made or bought by elderly people in preparation for death and kept in the attic. These coffins were made of *Tsuga chinensis*

**Fig. 18:**
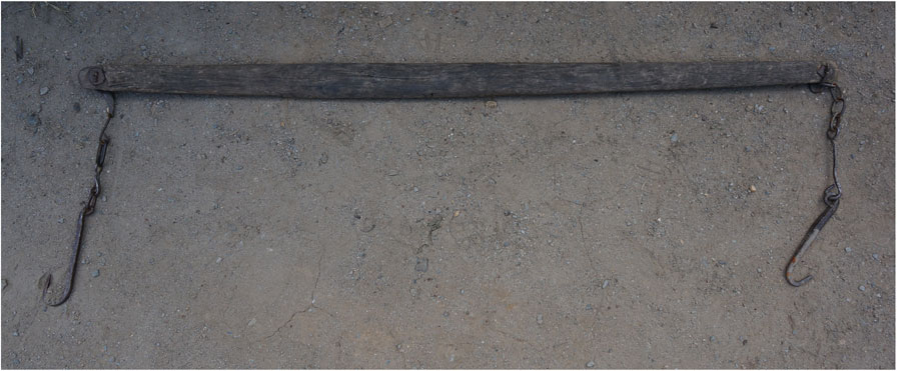
A *biandan* carrying stick made of *Morus australis* wood

**Fig. 19:**
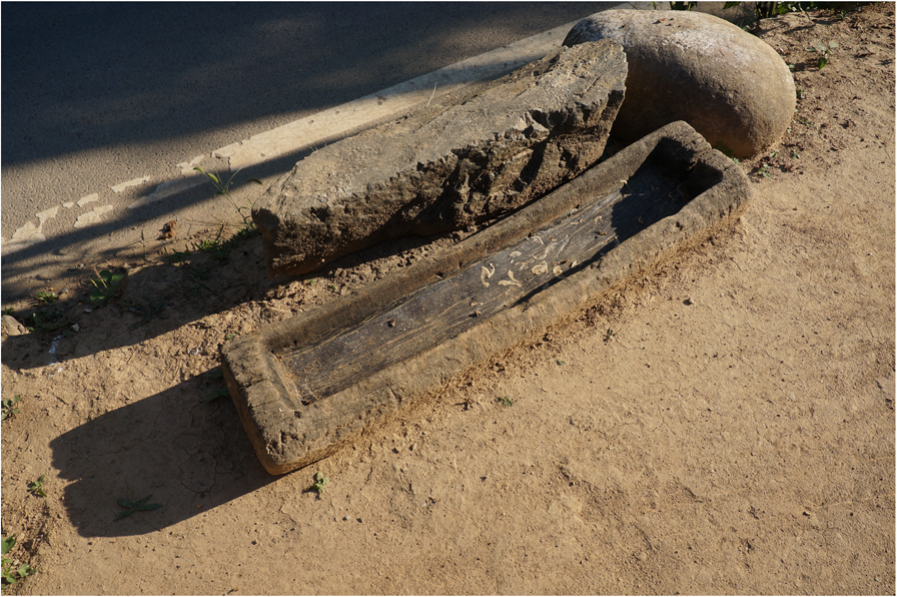
A trough for feeding farm animals made of *Castanea* wood

**Fig. 20:**
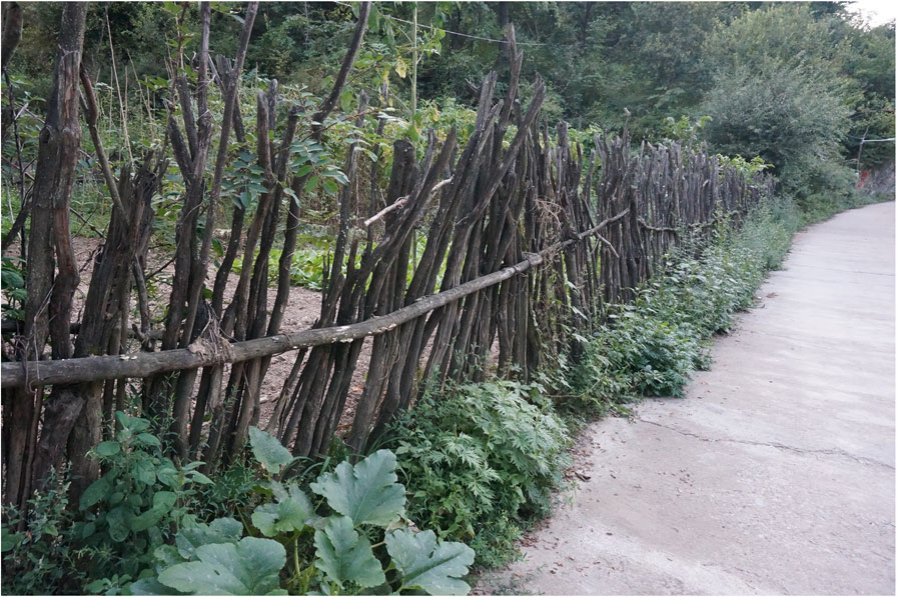
A ten-year old fence made from *Cotinus* sticks

**Fig. 21:**
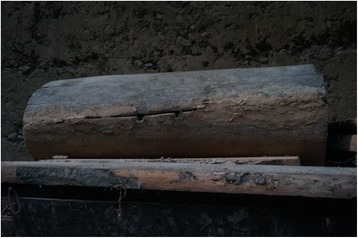
Traditional beehives are made of halved hollowed trunks of softwood deciduous trees (*Populus, Paulownia*)

**Fig. 22:**
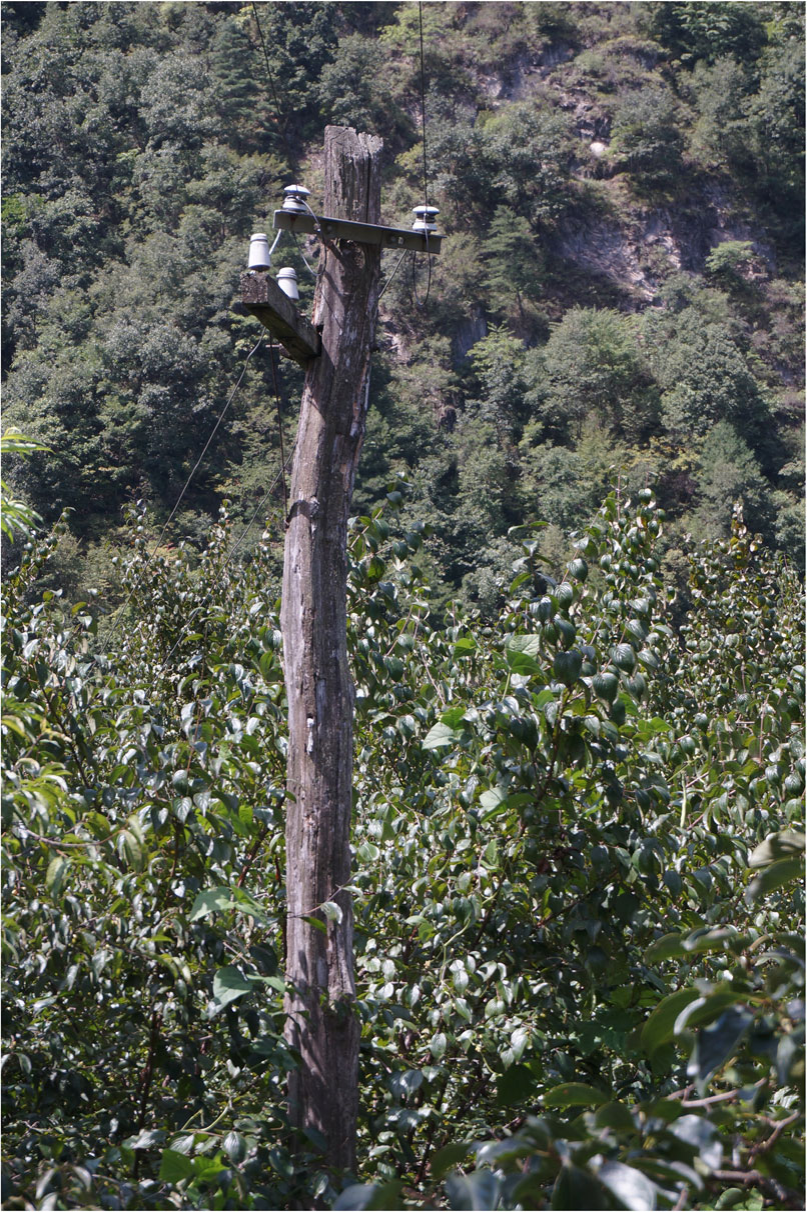
Up until recently electricity poles were made of *Castanea* trunks

**Fig. 23:**
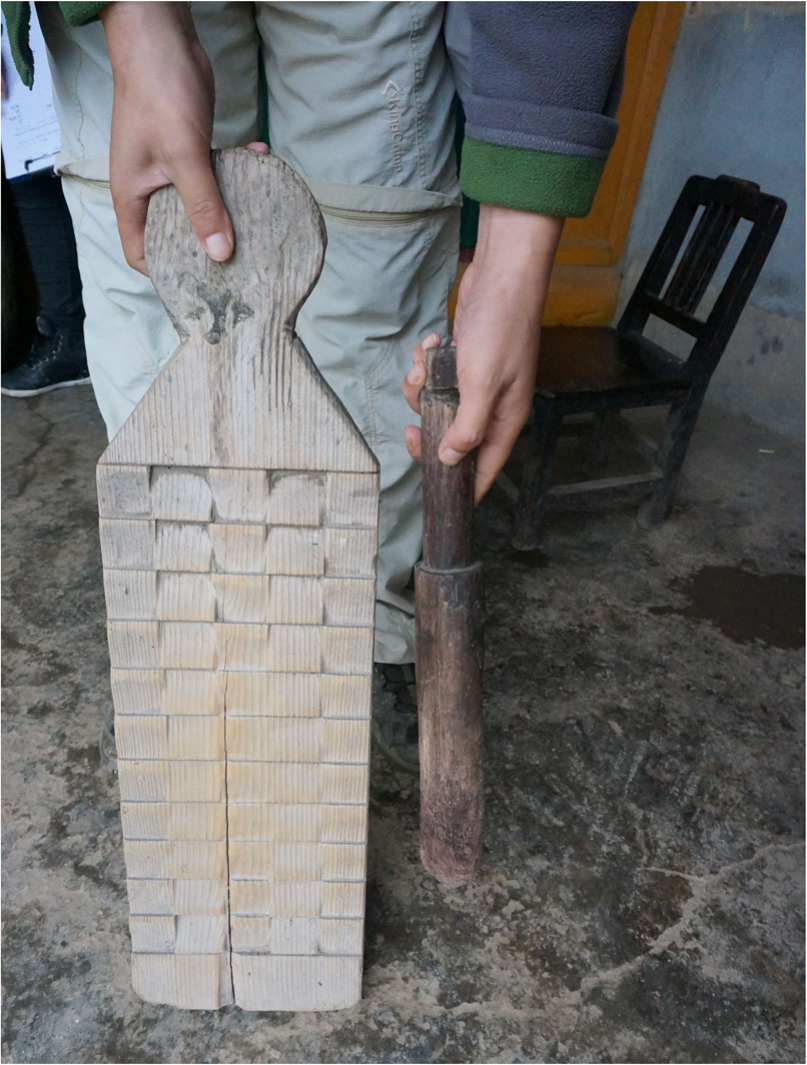
A washboard made of *Pinus tabuliformis* wood

The research was carried out following the code of ethics of the American Anthropological Association [26] and the International Society of Ethnobiology Code of Ethics [27]. Oral prior informed consent was acquired. The interviews were carried out in front of the dwellings of the interviewees in order to provide easy access to the tools and structures mentioned by the respondents. We asked the interviewees to list all the uses of wood, twigs or bark to make structures, tools and other objects in their own households and farms. This was the only question asked and at the beginning of the interviews no props were provided. At the end of each interview we asked to see the tools present in the yard, and sometimes more tree species were then mentioned (Figs. 2, 3, 4, 5, 6, 7, 8, 9, 10, 11, 12, 13, 14, 15, 16, 17, 18, 19, 20, 21, 22 and 23). Additionally, discussion groups were organized to cross-check the identification of specimens. The listed taxa (Tables 1, 2 and 3) were identified using specimens collected by informants in the forest or in the village. The interviews were carried out in Mandarin Chinese, which is the first language of the local population.

**Table 1:** The main emic categories of construction and tool plant use in the studied valley

Type of use	Use reports	Most preferred/used species
Furniture	92	*Prunus stellipila, Fraxinus mandshurica*
Construction	91	*Pinus tabuliformis, Pinus armandii*
Chopping boards	81	*Prunus stellipila, Betula albosinensis, Pyrus* sp.
Pick-axe handles	57	*Cornus kousa*
Spade handles	53	*Meliosma dillenifolia*
Doors	52	*Pinus tabuliformis, Pinus armandii*
Ladders	50	*Pinus armandii, Pinus tabuliformi,*
Carrying sticks	44	*Morus alba*
Beehives	42	*Populus purdomii, Paulownia tomentosa*
Shoes	41	*Tilia* spp.
Barrels	39	*Platycladus orientalis, Catalpa fargesii*
Tables	38	*Prunus stellipila*
Hoe handles	37	*Cornus kousa, Meliosma dillenifolia*
Coffins	34	*Tsuga chinensis*
Baskets	32	*Phyllostachys* spp., *Fargesia nitida*
Rolling pins	32	*Buxus sinica, Betula albosinensis, Cornus controversa, Stachyurus chinensis*
Walking sticks	31	*Philadelphus incanus*
Chairs	28	*Prunus stellipila*
Windows	28	*Pinus tabuliformis, Pinus armandii*
Firewood	22	*Quercus aliena*
Roof materials	18	*Cotinus coggygria*
Bridges	16	*Castanea mollissima*
Basket Handles	15	*Berchemia sinica*
Fences	13	*Castanea mollissima, Toxicodendron vernicifluum*
Ropes	12	*Pueraria montana* var. *lobata*
Grain shovels	12	*Salix* spp., *Pterocarya macroptera*
Fork handles	11	*Meliosma dillenifolia*
Harrow (teeth)	10	*Euonymus alatus*
Sickle handles	9	*Cornus kousa*
*Ciba* Hammers	9	*Eucommia ulmoides, Ulmus macrocarpa*
Ploughs	6	*Cornus* spp., *Quercus* spp.
Rake handles	4	*Cornus kousa*

**Table 2:** Most salient species freelisted by the interviewees

Latin name	Smith’s Salience Index
*Pinus tabuliformis* Carrière	35.5
*Pinus armandii* Franch.	27.9
*Prunus stellipila* Koehne	23.5
*Betula albosinensis* Burkill	18.5
*Cornus kousa* F.Buerger ex Hance	18.0
*Meliosma dilleniifolia* (Wall. ex Wight & Arn.) Walp.	17.0
*Fraxinus mandshurica* Rupr.	16.1
*Tsuga chinensis* (Franch.) Pritz.	13.6
*Populus purdomii* Rehder	11.8
*Catalpa fargesii* Bureau	11.5
*Quercus aliena* var. *acutiserrata* Maxim.	11.1
*Morus australis* Poir.	10.8
*Castanea mollissima* Blume	10.4
*Toona sinensis* (Juss.) M.Roem.	9.6
*Tilia olivieri* Szyszył. and *T. paucicostata* Maxim.	9.4
*Ailanthus altissima* (Mill.) Swingle	9.0
*Populus cathayana* Rehder	9.0
*Platycladus orientalis* (L.) Franco	8.9
*Phyllostachys* sp.	8.5
*Cornus controversa* Hemsl.	7.7

**Table 3:** The list of species used for construction, furniture and other handicrafts

Latin name	Local name	Local name (Chines characters)	No. of citations	Part	Use	Voucher numbers, begin with WUK Kang
*Pinus tabuliformis* Carrière	songmu	松木	37	wood	house construction esp. roofs, furniture, ladders, beehives	K198
*Prunus stellipila* Koehne	kutao	苦桃	34	wood	mainly furniture and chopping boards	K101,103
*Pinus armandii* Franch.	madengsong	马灯松	31	wood	house construction, furniture, doors, windows, ladders	K157
*Betula albosinensis* Burkill	honghua, huamu,	红桦,桦木	30	wood and bark	wood for chopping boards, stools, also rolling pins; bark for hats and steamers	K164
*Fraxinus mandshurica* Rupr.	shuiquliu	水曲柳	30	wood	highly valued for furniture, also window frames, handles (esp. spades), carrying sticks etc.	K140
*Castanea mollissima* Blume	maoli	毛栗	27	wood	best for electricity posts and for boards in bridges, also pig troughs, roof elements, door frames	k132
*Cornus kousa* F.Buerger ex Hance	shizao	石枣子	26	wood	handles (axe, hoe, sickle), also rolling pins and stone grinder axes, and firewood	K155
*Meliosma dilleniifolia* (Wall. ex Wight & Arn.) Walp.	linshu, xiangnongmu	林寿,降龙木	26	wood	highly valued for handles (esp. hoe, spade and rake) - very smooth and durable	K118
*Populus purdomii* Rehder	dongguayang, baiyang, yangshu	冬瓜杨,白杨,杨树	26	wood	mainly for bee-hives, also “cuopiao” grain shovels	K180
*Tilia olivieri* Szyszył. and *T. paucicostata* Maxim.	duanmu, duanshu	椴木(树)	26	bark and wood	mainly bark for shoes, also wood for beehives, ladders, musical instruments, boxes and furniture	K184, K187
*Catalpa fargesii* Bureau	tangqiu	唐楸	23	wood	mainly for barrels, also for furniture due to its attractive texture and grain	K113
*Phyllostachys* sp.	shuizhu, jinzhu, banzhu, zhuzi	水竹,金竹,斑竹,竹子	23	above ground parts	mainly baskets, also basket handles, fishing rods, washing up brushes	K133, K134
*Quercus aliena* var. *acutiserrata* Maxim.	gangmu	杠木	23	wood	construction esp. for beams, pillars, floor boards; best for firewood, also handles and “muer” mushroom cultivation	K181
*Tsuga chinensis* (Franch.) Pritz.	zaosong	枣松	22	wood	mainly coffins and furniture, also construction (eg roof rafters) and barrels	K148
*Morus australis* Poir.	sangmu	桑木	21	wood	carrying sticks	K156
*Philadelphus incanus* Koehne	jigutou	鸡骨头	18	wood	walking sticks	K163
*Ailanthus altissima* (Mill.) Swingle	baichun	白椿	17	wood	furniture, esp. boards for windows and doors, also for table and chair legs and chopping boards	K177
*Platycladus orientalis* (L.) Franco	baimu, xiangbai	柏木,香柏	17	wood	mainly water barrels and containers, and coffins	K121
*Toona sinensis* (Juss.) M.Roem.	hongchun	红椿	17	wood	mainly for furniture, windows and door planks	K144
*Cornus controversa* Hemsl.	liangzimu	梁子木	15	wood	hard wood for chopping boards, furniture legs and rolling pins, also for handles	K109, K128
*Pyrus* sp.	limu	梨木	15	wood	mainly chopping boards	K165
*Quercus variabilis* Blume	xiangmu, xiangshu	橡木(树)	15	wood and bark	best for firewood, handles (basket, axe, plough), bark for industry and shoe soles, “muer” mushroom cultivation, furniture, boards	K160
*Berchemia sinica* C.K.Schneid	yagutiao	牙骨条	14	wood	mainly for walking sticks, cattle harnesses and basket handles, also pitch-fork fingers	K126
*Cotinus coggygria* Scop.	huanglou	黄栌	14	wood	mainly for roof elements supporting tiles, also for “mu” harrows and fence posts	K119
*Magnolia sprengeri* Pamp.	jiangbo, mubieshu	姜剥	14	wood	mainly for high quality chopping boards	K138
*Paulownia tomentosa* Steud.	tongmu	桐木	14	wood	mainly for beehives, also barrels, pot covers, ladders, coffins and low-weight boards	K170
*Toxicondendron vernicifluum* (Stokes) F.A. Barkley	qimu, qishu	漆木(树)	14	wood and secretion	mainly for electricity posts, also for barrels, fences, boards under tiles, stem sap used for lacquer	K152
*Pueraria montana* var. *lobata* (Willd.) Sanjappa & Pradeep	geteng, getiao	葛藤,葛条	13	wood	fibre for shoes, ropes and baskets	K143
*Fargesia nitida* (Mitford) Keng f. ex T.P.Yi	songhuazhu, zhuzi	松花竹,竹子	12	above-ground parts	brooms and baskets	K188
*Populus cathayana* Rehder	baiyang, yangmu	白杨,杨木	12	wood	mainly construction material and ladders, also fence posts, troughs and shovels	K127
*Abies fargesii* Franch.	pumu, pusong	朴木,朴松	11	wood	construction, coffins, ladders	K125
*Salix* sp.	liu, liumu, liutiao	柳,柳木,柳条	10	wood, year-old twigs, bark	shovels, twigs for baskets, bark for shoes, also firewood	K158
*Cornus officinalis* Siebold & Zucc.	zaopi	枣皮	9	wood	mainly handles (spades, axes) and firewood, also ploughs	K189
*Pterocarya macroptera* Batalin	maliu	麻柳	9	wood and bark	wood, mainly shovels and dustpans, bark for making shoes	K147
*Ulmus macrocarpa* Hance	yumu	榆木	9	wood	a variety of small objects: “ciba” hammers, “mu” harrows, ladders, basket handles, ploughs, furniture	K174
*Amelanchier sinica* (C.K.Schneid.) Chun	hongshenzi, hongshunzi	红绳子,红顺子	8	wood	mainly handles (for hoe, axe, rake), baskets	K106
*Juglans regia* L.	hetao	核桃	8	wood	furniture, feet of door frames	K137
*Quercus spinosa* David	tiejiamu	铁匠木	8	wood	mainly for handles (hoe, axe), wooden hammers, rolling pins, axes of stone grinders, firewood	K159
*Symplocos paniculata* (Thunb.) Miq.	baihuacha	百花茶	8	wood	handles (sickle, hoe, axe, spade)	K131
*Buxus sinica* (Rehder & E.H.Wilson) M.Cheng	huangyang	黄杨	7	wood	very hard wood, the best material for rolling pins and carving elements of Chinese board games “xianqi” and “majiang”	K167
*Eucommia ulmoides* Oliv.	duzhong	杜仲	6	wood	a very good handle for hoes, material for “ciba” hammers	K190
*Fraxinus platypoda* Oliv.	baixingmu	白芯木	6	wood	handles (axe, spade), also furniture esp. legs	K117
*Sorbus folgneri* (C.K.Schneid.) Rehder	baishenzi	白绳子	6	wood	handles (hoe, axe, spade)	K108
*Acer stachyophyllum* Hiern (syn. *Acer tetramerum* Pax)	hongliu	红柳	5	wood	handles, esp. hoe, spade and pick-axe	K175
*Juglans mandshurica* Maxim.	mahetao	麻核桃	5	bark	bark for shoes and ropes	K191
*Picea wilsonii* Mast.	zimu	紫木	5	wood	construction and coffin boards	K124
*Stachyurus chinensis* Franch.	tonghuagan	通花杆	5	wood	mainly rolling pins, also arms of scales, walking sticks, instruments for blowing fire	K149
*Cannabis sativa* L.	huoma	火麻	4	annual above ground parts	fibre for shoes and ropes	K192
*Prunus tomentosa* Thunb.	chuantao	川桃	4	wood	“lianjia” flails, basket handles, firewood	K161
*Bassia scoparia (L.) A.J.Scott*	saozhoucai	扫帚菜	*3*	above-ground parts	brooms	
*Betula luminifera* H.J.P.Winkl.	miaoyumu	描榆木	3	wood	carrying sticks, firewood	K120
*Broussonetia papyrifera* (L.) L’Hér. ex Vent.	goushu	构树	3	bark	bark for shoes and ropes	K176
*Juniperus chinensis* L.	baishu, yabei	柏树,崖柏	3	wood	decorative roots (“gendiao”), body ornaments due to pleasant smell, furniture	K129
*Lonicera standishii* Jacques	jigutou, paoer, yangnaishu	鸡骨头,泡儿,羊奶树	3	wood	walking sticks, handles (sickle, axe), rolling pins	K145
*Maackia hupehensis* Takeda	chouhuai, honghuai	臭槐,红槐	3	wood	ladders, stools, handles (wheel barrows)	K182
*Prunus davidiana* (Carriere) Franch.	shantao	山桃	3	wood	chopping boards, rolling pins, branches to drive ghosts away	K169
*Viburnum betulifolium* Batalin	cusuantiao, nuomitiao	苦酸条,糯米条	3	wood	axe handles, rolling pins, rakes, “mu” harrows	K142
*Corylus heterophylla* Fisch. ex Trautv.	zhenzi	榛子	2	wood	handles (hoe, axe), frames for garden climbers	K171
*Euonymus alatus* (Thunb.) Siebold	bashu	巴树(木)	2	wood	“mu” harrows	K193
*Kalopanax septemlobus* (Thunb.) Koidz.	ciqiu	刺楸	2	wood	furniture	K122
*Paederia foetida* L.	hongteng, jishiteng	红藤,鸡屎藤	2	wood	baskets, ropes	K194
*Rhododendron* sp.	doujuan, pipa	杜鹃,枇杷	2	wood	rolling pins, “xiba” washing sticks	K173
*Rhus potaninii* Maxim.	wubeizi	五倍子	2	wood	electricity posts, barrels	K151
*Sophora japonica* L.	huaimu, huaishu	槐木(树)	2	wood	furniture, chopping boards	K183
*Taxus wallichiana* var. *chinensis* (Pilg.) Florin	hongdoushan	红豆杉	2	wood	barrels and containers for water	K123
*Viburnum schensianum* Maxim.	heichagun	黑茶棍	2	wood	mainly wooden fork fingers, also basket handles, “mu” harrows, “lianjia” flails	K179
*Akebia trifoliata* (Thunb.) Koidz.	mutong	木通	1	wood	ropes	
*Betula platyphylla* Sukachev	huashu	桦树	1	wood	firewood	K153
*Caragana arborescens* Lam.	yangqiuhua	洋秋花	1	wood	brushes for cleaning kitchen pots, “mu” harrow teeth	K186
*Cephalotaxus sinensis* (Rehder & E.H.Wilson) H.L.Li	shubai	水柏	1	wood	basket handles	K195
*Chaenomeles sinensis* (Dum.Cours.) Koehne	muguahaitang	木瓜海棠	1	wood	walking sticks	k196
*Crataegus cuneata* Siebold & Zucc.	yeshanza	野山楂	1	wood	chopping boards, table legs	K197
*Dipteronia sinensis* Oliv.	shanmagan	山麻杆	1	wood	big barrels for water and alcohol fermentation	K116
*Elaeagnus umbellata* Thunb.	jianzici	剪枝刺	1	wood	pitch-forks	K136
*Juniperus squamata* Buch.-Ham. ex D.Don	yabei	崖柏	1	wood	big barrels for water or spirits	K199
*Larix gmelinii* var. *principis-rupprechtii* (Mayr) Pilg.	luoyesong	落叶松	1	wood	boards	K135
*Ligustrum* sp.	duijetiao	对节条	1	wood	fork fingers	K162
*Malus pumila* Mill.	pingguoshu	苹果树	1	wood	“ciba” hammers	K166
*Miscanthus sinensis* Andersson	maocao	茅草	1	wood	roof thatching	K141
*Prunus* sp.	choutao	臭桃	1	wood	chopping boards	K102
*Rhus chinensis* Mill.	fulianzi	伏莲子	1	wood	charcoal for fireworks	K172
*Sorbaria kirilowii* (Regel & Tiling) Maxim.	gaolianggan	高粱杆	1	wood	rolling pins	K185
*Vitex negundo* L.	huangjintiao	黄荆条	1	wood	basket handles	k178

The authorities of Houzhenzi Forest farm in Shaanxi Forestry Bureau in Xi’an and park rangers were also consulted about the conservation status of trees in the study area.

In order to measure the cultural importance of particular wild foods we used Smith’s Salience Index [28]. The index for species A is the mean of the following ratio calculated for each free listed plant:

$$ \mathrm{Salience}\kern0.5em \mathrm{Index}\kern0.5em =\kern0.5em \frac{\mathrm{total}\kern0.5em \mathrm{no}.\kern0.5em \mathrm{of}\kern0.5em \mathrm{item}\mathrm{s}\kern0.5em \mathrm{in}\kern0.5em \mathrm{a}\kern0.5em \mathrm{list}\hbox{-} \operatorname{rank}\kern0.5em \mathrm{order}\kern0.5em \mathrm{of}\kern0.5em \mathrm{s}\mathrm{pecies}\kern0.5em \mathrm{A}\kern0.5em \left(\mathrm{starting}\kern0.5em \mathrm{from}\kern0.5em 0\kern0.5em \mathrm{for}\kern0.5em \mathrm{the}\kern0.5em 1\mathrm{st}\kern0.5em \mathrm{item}\right)}{\mathrm{total}\kern0.5em \mathrm{number}\kern0.5em \mathrm{of}\kern0.5em \mathrm{item}\mathrm{s}\kern0.5em \mathrm{in}\kern0.5em \mathrm{a}\kern0.5em \mathrm{list}} $$

Thus a species which is always quoted first gets an index which equals 1 and the items quoted at the end of the freelists tend to have Smith’s indexes close to 0.

Voucher specimens of plants were deposited in the Herbarium of the Northwest A&F University in Yangling (WUK). Plants were identified using the standard identification key concerning local floras, and their names follow the Plant List [29].

## Results

Altogether, 84 species of plants were recorded as material for construction and handicraft plants (Tables 1, 2 and 3). Of these, 80 species are used for their wood and five species for bark. Two herbaceous species and two bamboo taxa were used (Table 3). The most frequently mentioned plants were: *Pinus tabuliformis* Carrière, *Prunus stellipila* Koehne, *Pinus armandii* Franch., *Betula albosinensis* Burkill, *Fraxinus mandshurica* Rupr., *Castanea mollissima* Blume, *Cornus kousa* F.Buerger ex Hance, *Meliosma dilleniifolia* (Wall. ex Wight & Arn.) Walp., *Populus purdomii* Rehder, *Tilia olivieri* Szyszył. and *T. paucicostata* Maxim. (Table 3). The ranking of most salient species is nearly identical to that of those most frequently mentioned (Table 2).

Both the mean and median number of species mentioned per interview was 22.

All the large-sized tree species are used in some form by the local inhabitants. Among shrubby species and small trees those which have very hard wood are used to make handles, walking sticks or small objects like forks and harrow teeth. The bark of a few species was used to make shoes, hats, steamers and ropes, but this tradition is nearly gone. A few species, mainly bamboo, are used for basket making and year-old willow branches are used for brushing off the chaff during wheat winnowing. The use of large pieces of local timber has greatly diminished due to the protection regime, and is now limited to the trees growing in the land around houses. On the other hand, the wood for such objects as tool handles, bee hives, walking sticks and carrying sticks is still commonly used from local trees.

We recorded a few dozen emic categories of use. The most frequently mentioned categories were listed in Table 1. A few of the most commonly used tree species have many uses, but among the trees used with medium frequency some have very specialized uses restricted to one particular application. For example *Morus australis* is the preferred wood for carrying sticks (a stick where two buckets are attached on each side), *Philadelphus incanus* for making walking sticks, *Castanea mollissima* for electricity poles, *Cotinus coggygria* for making small boards supporting ceramic tiles in the roof, *Tsuga chinensis –* coffins, *Meliosma dillenifolia* and *Cornus kousa* – tool handles. *Pinus* spp. is used for the main construction of houses, windows and doors. The materials for making chopping boards and rolling pins are more diverse, though for the former *Prunus stellipila* and for the latter *Buxus sinica* is preferred. Firewood is usually collected from any available wood, though *Quercus* and *Betula* are preferred.

All the households contain many self-made wooden tools. These tools are usually made only for farmers’ use and are neither bought or sold. Such items as furniture, coffins, handles or shovels are still commonly made. On the other hand the manufacturing of bark shoes disappeared in the 1980s and we could not find a single such shoe preserved in the valley, although many people still know how to make them. The production of wooden barrels is also dying out.

## Discussion

It is difficult to compare our data with other places in China as similar studies are lacking.

One of the factors which makes the sale of wooden items hard, even for those skilled in making them, is the protection status of the surrounding forest. No commercial large scale logging has been performed in the area since 1987, when it was designated as a water resource area for the city of Xi’an. Wood is only cut for local purposes for farmers’ use. The monitoring of timber use is important for forest conservation [30–32]. Our results suggest that some rare and endangered tree species may have been selectively cut by local people due to their valuable wood, e.g. *Fraxinus mandshurica* and *Taxus wallichiana* var. *chinensis*. Some other rare species, e.g. *Dipteronia sinensis,* are little used and little valued.

All the local large canopy trees are used for some purpose. From among smaller trees and shrubs, those which are particularly hard are selectively cut. From all the larger trees more common in the area, *Pterocarya* is used the least. It is also striking that only one species of *Acer* was mentioned, although a few other species of this genus grow in the forests. They tend, however, to grow above the villages, at slightly higher altitudes, and they are not attractive due to their shrubby growth. Some other common shrubs, like *Spiraea* were not mentioned either.

The use of smaller tree species is also very common, as they usually grow on farmer’s parcels. According to regulation no. 32 in chapter 5 of the “State Forest protection Laws,” [33] private trees in farmers’ parcels around their dwellings can be utilized by local residents, even in National Forest Parks. For example, local people planted a plantation of *Cornus officinalis* on their own land for money, but in recent years the price of the fruit of this species has become very low. So many people felled the *C. officinalis* plantations and the wood was used to make tools or firewood. According to the information we got from the nature conservation authorities local residents occasionally get permission to cut *Castanea* trees in the state part of the forest for the construction of bridges, whereas construction timber is now imported from outside the park borders. The demand for construction timber has also been diminished by the use of non-wooden construction materials (e.g. concrete). Some wood is also available to local residents as a leftover from forest management (e.g. removing trees attacked by pests).

It is very striking that hardly any superstitious beliefs were recorded when talking about trees. No trees were treated as particularly lucky (auspicious) or unlucky, as is very common in other parts of the world [34], and despite the presence of such beliefs in the traditional *fengshui* system [35].

Although some plant uses are well known, probably across large parts of China, particularly those concerning large hardwoods used for construction and furniture, or bamboo (see e.g. [36], some uses of rarer small trees and shrubs in handicrafts may be endemic to this part of China, and be worth recording.

## Conclusions

The high diversity of woody species facilitates the preservation of rich knowledge about the properties of many lesser known kinds of wooden materials. In spite of social changes, some tools and utensils are still handmade (handles, chopping boards, furniture), whereas other handicrafts have completely disappeared (bark shoes, ropes) or are disappearing (barrels). Generally, the impact of these activities on the tree population is probably very low.
